# Social and Poor Law Problems

**Published:** 1907-01-05

**Authors:** 


### SOCIAL AND POOR LAW PROBLEMS.

### THE TEACHING OF THE MENTALLY DEFECTIVE.

The education of the mentally defective seemed, when it was begun comparatively few years ago, one of the most hopeless of experiments. It is possible that in many cases they were withdrawn from the ordinary schools where they had hitherto been sent, as much because their presence was a hindrance to their companions of normal mentality as because it was thought that special training would do them good. But, according to a report recently brought out by Mr. R. Blair, the executive officer of the County Council, on the special schools in London, the results of the work have been eminently satisfactory. In many cases what has been regarded as mental defect may have been only slowness of development, for we are informed that after some instruction in the special schools no fewer than 126 children were able to return to the ordinary schools, while during the past year 131 out of the 159 children between the ages of 14 and 16 who left the schools during the past year, were able to find work at wages ranging from one to fifteen shillings a week. The number of children who did not leave until they were 16 years of age was 92, and of these 38 obtained work. This is on the face of it encouraging, but without knowing what was the nature of the employment which the children got, we cannot be quite certain that this means a marked degree of mental improvement. The putting of children of weak mind to work is no new thing; it was one of the features of the early factory system, and though much admired by the philosophers of the early nineteenth century, has failed to commend itself to later generations. It certainly is desirable, for their own sakes as well as for that of their families, that defectives should be self-supporting, but as their weakness makes them peculiarly a prey to tyranny, more care would need to be exercised in placing them than if they were of normal intellect. This only means that after-care is as important as education. Thus, while domestic service is a good thing for normal girls, it is suited for defective girls only under circumstances of protection and consideration not easily obtainable. Defective boys who are of strong physique are likely to find work as labourers as easily as their more intelligent brethren, but when, as often happens, both mind and body are weak, suitable employment is more difficult to find. This is, however, no reason for giving up the defective as hopeless, but rather for continuing after school days are over as much as possible of the care that then improved their condition so much.

